# Validation: The Use of Google Trends as an Alternative Data Source for COVID-19 Surveillance in Indonesia

**DOI:** 10.1177/1010539520940896

**Published:** 2020-07-09

**Authors:** Ricvan Dana Nindrea, Nissa Prima Sari, Lutfan Lazuardi, Teguh Aryandono

**Affiliations:** 1 Universitas Gadjah Mada, Yogyakarta, Indonesia; 2 Universitas Andalas, Padang, Indonesia

The utilization of the Google search engine will construct digital traces, which can be a worthy source of information, and it has been rapidly used for health-related purposes in recent years. In the current COVID-19 outbreak, the use of digital traces might be helpful in epidemiological identification. The use of digital traces, otherwise known as digital epidemiology, is a new field for exploring disease patterns and changes in health dynamics within a population by utilizing digital traces.

## Google Trends and COVID-19 Surveillance in Indonesia

The COVID-19 affected 215 countries, and 190 countries confirmed local transmission.^1^ Global data showed that there have been 5 304 772 positive confirmed cases, and 342 029 cases have resulted in death due to COVID-19 with a mortality rate of 6.4%. In addition to this global conditions, Indonesian data on May 26, 2020, reported 22 750 confirmed as infected and 1391 deaths with a mortality rate of 6.1%.^2^

COVID-19 was a newly discovered disease; therefore, knowledge related to its infection prevention and control measures is still limited.^2^ In this regard, almost all societies in Indonesia demand information related to COVID-19, which includes symptoms, transmission, and treatment by utilizing various media, both electronic and print. One of the most widely used media is to use search engines, and Google search engine is the most popular.^3^

The growth of internet usage is rapidly increased along with the widespread penetration of cellular phones and the development of artificial intelligence. Therefore, this condition provides a promising opportunity for the development of digital epidemiology in contributing and supporting the health system in general and national surveillance of COVID-19 in particular.^4,5^ The use of this field can potentially help the discrepancy in conventional surveillance systems in developing countries where the report often constrained from limited timeliness, underreporting, or limited budget to support the needs of infrastructures.^5^

Digital data recorded on Google can be traced through the Google Trends website (https://trends.google.com/trends/). Previous studies found that Google Trends data are highly associated with traditional surveillance data.^5,6^ Previous studies explained the benefits of using Google Trends in the early phase, which are easy and cost-effective compared with conventional reporting systems. However, the application of Google Trends is still lacking as it is influenced by several media clamors rather than the actual epidemiological burden that must be reported. Indonesia has an overall internet usage of 54.7%, and the utilization of Google is reported to be considerable at 98.0%; hence, this also demonstrates a great opportunity to harness Google Trends in Indonesia.^7^
